# Supplementary figures and images for: Responses of rhizosphere bacterial communities with different niche breadths to liquid fertilizer produced from Fuji apple wastes during planting process
Source: Microbiol Spectr. 2025 May 30;13(7):e02068-24. doi: 10.1128/spectrum.02068-24 (PMC12211022; doi:10.1128/spectrum.02068-24)

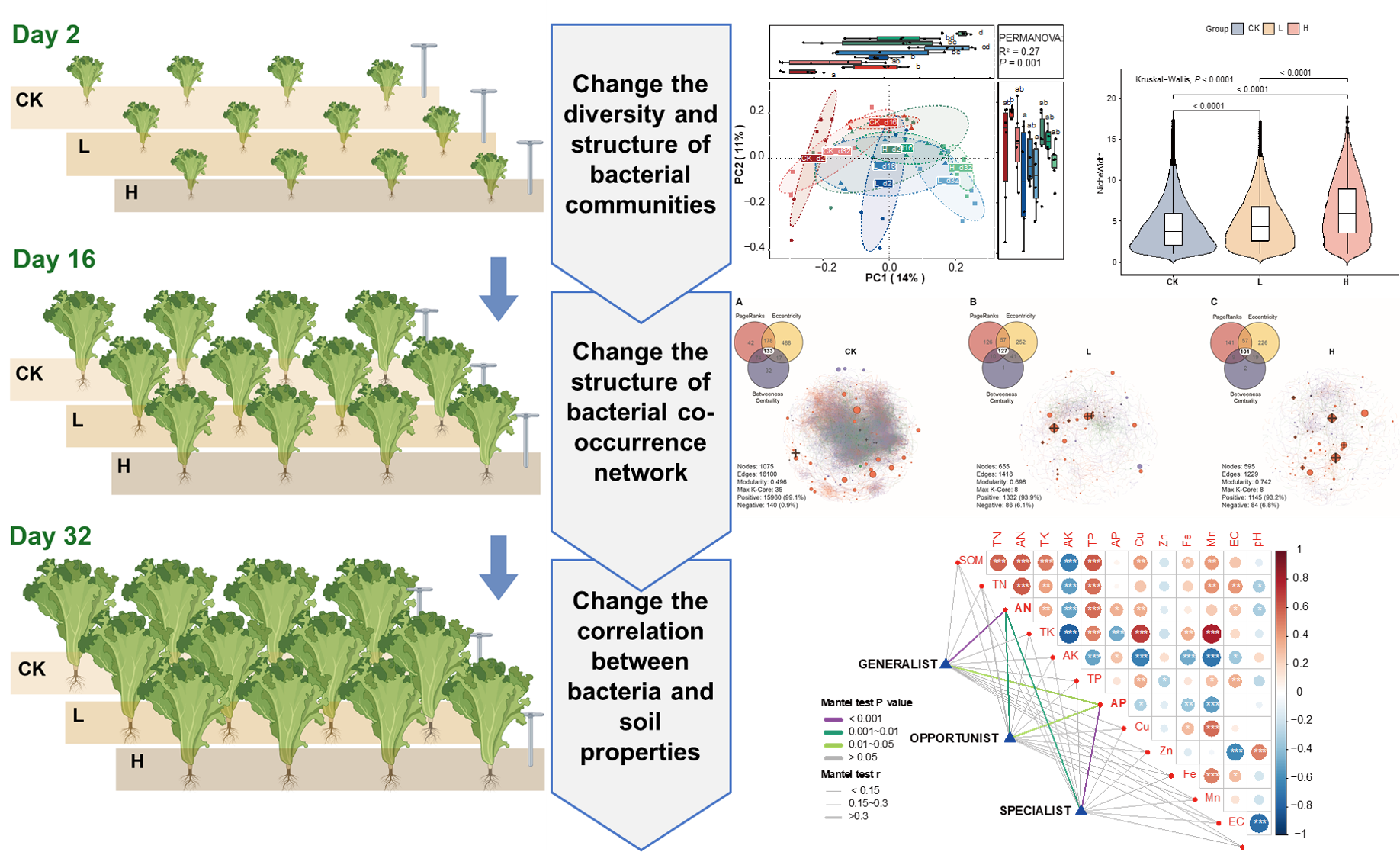

Supplement: Fig. S1 — Graphic abstract. [file spectrum.02068-24-s0001.tif]
