# Supplementary material for: Responses of rhizosphere bacterial communities with different niche breadths to liquid fertilizer produced from Fuji apple wastes during planting process
Source: Microbiol Spectr. 2025 May 30;13(7):e02068-24. doi: 10.1128/spectrum.02068-24 (PMC12211022; doi:10.1128/spectrum.02068-24)
Supplement: Supplemental tables — Tables S1 to S7. [file spectrum.02068-24-s0002.docx]

**Table S1** Alpha indices of bacterial community in the study. All values were an average from all replicates ± standard deviations (SD).

| **Groups** | **Days** | **Chao1** | **Shannon** | **Faith PD** | **Good coverage** |
| --- | --- | --- | --- | --- | --- |
| **CK** | **2** | 6271.113±1715.567 | 7.285±0.903 | 357.624±82.081 | 0.983±0.004 |
|  | **16** | 10248.522±1720.338 | 8.067±0.067 | 459.387±17.146 | 0.961±0.015 |
|  | **32** | 6928.006±3074.215 | 7.568±0.574 | 382.932±83.897 | 0.979±0.013 |
| **L** | **2** | 8061.217±3084.731 | 7.007±1.095 | 410.794±78.804 | 0.971±0.014 |
|  | **16** | 8400.841±2929.293 | 7.724±0.354 | 418.889±65.369 | 0.97±0.018 |
|  | **32** | 9823.701±1187.183 | 7.56±0.474 | 443.571±19.437 | 0.961±0.012 |
| **H** | **2** | 9653.49±3082.299 | 7.764±0.531 | 439.574±65.144 | 0.962±0.018 |
|  | **16** | 10278.691±1634.74 | 7.872±0.334 | 470.05±24.116 | 0.963±0.012 |
|  | **32** | 11244.7±404.442 | 7.88±0.211 | 461.99±10.038 | 0.952±0.004 |

**Table S2** Bray−Curtis dissimilarity, βNTI and niche breadths of bacterial community in the study. All values were an average from all replicates ± standard deviations.

| **Groups** | **Days** | **Bray−Curtis dissimilarity** | **βNTI** | **Niche Breadth** |
| --- | --- | --- | --- | --- |
| **CK** | **2** | 0.702±0.161 | 3.029±1.490 | 1.949±0.969 |
|  | **16** | 0.442±0.076 | 4.948±1.906 | 3.104±1.287 |
|  | **32** | 0.662±0.117 | 3.045±1.180 | 2.052±1.006 |
| **L** | **2** | 0.659±0.132 | 2.311±1.495 | 2.297±1.031 |
|  | **16** | 0.597±0.101 | 3.167±1.709 | 2.305±1.065 |
|  | **32** | 0.49±0.07 | 3.389±1.299 | 2.802±1.290 |
| **H** | **2** | 0.548±0.207 | 4.964±3.022 | 2.645±1.184 |
|  | **16** | 0.506±0.069 | 3.806±2.280 | 2.852±1.227 |
|  | **32** | 0.367±0.058 | 1.571±1.890 | 3.248±1.415 |

**Table S3** Relative abundance of different groups and different niche breadths in the top 10 phyla and the top 15 genera.

| **Niche breadth** | **Species taxonomic level** | **CK (%)** | | | **L (%)** | | | **H (%)** | | |
| --- | --- | --- | --- | --- | --- | --- | --- | --- | --- | --- |
|  |  | **Day2** | **Day16** | **Day32** | **Day2** | **Day16** | **Day32** | **Day2** | **Day16** | **Day32** |
|  | **Phylum** |  |  |  |  |  |  |  |  |  |
| **SPECIALIST** | Alphaproteobacteria | 11.21 | 13.05 | 12.21 | 18.52 | 14.21 | 19.18 | 15.35 | 13.43 | 18.63 |
|  | Gammaproteobacteria | 9.33 | 6.85 | 8.07 | 15.05 | 9.35 | 7.77 | 8.24 | 10.42 | 9.39 |
|  | Actinobacteria | 7.83 | 5.35 | 7.55 | 7.12 | 7.29 | 9.44 | 5.22 | 5.05 | 6.18 |
|  | Acidobacteria | 9.18 | 7.71 | 7.63 | 4.66 | 5.52 | 4.48 | 7.19 | 6.57 | 4.74 |
|  | Bacteroidetes | 4.24 | 4.27 | 6.17 | 4.23 | 5.4 | 5.46 | 5.3 | 3.63 | 5.2 |
|  | Planctomycetes | 7.63 | 3.85 | 6.72 | 4.35 | 4.59 | 2.51 | 3.96 | 4.8 | 1.66 |
|  | Deltaproteobacteria | 4.68 | 4.48 | 5.51 | 3.38 | 5.7 | 3.19 | 3.12 | 4.51 | 3.14 |
|  | Betaproteobacteria | 5.26 | 2.75 | 4.2 | 2.77 | 3.65 | 3.7 | 3.96 | 2.97 | 4.73 |
|  | Firmicutes | 2.82 | 1.39 | 2.22 | 5.72 | 4.04 | 4.1 | 2.35 | 3.15 | 2.31 |
|  | Verrucomicrobia | 1.6 | 0.97 | 1.22 | 1 | 1.33 | 0.91 | 0.74 | 1.04 | 0.74 |
|  | Others | 5.61 | 2.17 | 3.67 | 2.67 | 3.09 | 2.58 | 2.93 | 2.77 | 2.49 |
| **OPPORTUNIST** | Acidobacteria | 6 | 9 | 6.42 | 5.09 | 5.9 | 5.52 | 8.33 | 7.23 | 6.02 |
|  | Alphaproteobacteria | 3.19 | 5.52 | 4.07 | 3.81 | 4.25 | 4.87 | 4.89 | 4.59 | 5.73 |
|  | Planctomycetes | 4.9 | 4.57 | 4.65 | 3.9 | 4.42 | 3.05 | 3.76 | 5.23 | 2.33 |
|  | Actinobacteria | 2.54 | 4.6 | 2.79 | 2.41 | 3.07 | 3.58 | 3.57 | 3.11 | 4.06 |
|  | Betaproteobacteria | 1.97 | 3.31 | 2.42 | 2 | 2.39 | 2.72 | 2.95 | 2.68 | 3.18 |
|  | Gammaproteobacteria | 1.71 | 2.33 | 1.74 | 1.57 | 1.79 | 1.55 | 1.91 | 1.99 | 1.87 |
|  | Deltaproteobacteria | 1.12 | 2.15 | 1.63 | 1.27 | 1.65 | 1.53 | 1.84 | 1.94 | 1.96 |
|  | Firmicutes | 1.22 | 1.62 | 1.37 | 1.37 | 1.63 | 1.65 | 1.55 | 1.92 | 1.41 |
|  | Bacteroidetes | 0.8 | 1.48 | 1.08 | 1.04 | 1.26 | 1.56 | 1.16 | 1.26 | 1.95 |
|  | Verrucomicrobia | 0.63 | 1.19 | 0.9 | 0.75 | 0.96 | 1.07 | 0.94 | 1.23 | 1.31 |
|  | Others | 1.76 | 3.1 | 2.39 | 1.91 | 2.39 | 2.6 | 3.07 | 3.06 | 2.9 |
| **GENERALIST** | Actinobacteria | 0.75 | 1.48 | 0.73 | 0.78 | 0.93 | 1.17 | 1.22 | 1.04 | 1.38 |
|  | Acidobacteria | 0.62 | 1.34 | 0.79 | 0.78 | 0.89 | 0.97 | 1.3 | 1.19 | 1.16 |
|  | Firmicutes | 0.78 | 0.96 | 0.9 | 0.8 | 0.9 | 0.98 | 0.93 | 0.97 | 0.98 |
|  | Alphaproteobacteria | 0.48 | 0.97 | 0.55 | 0.63 | 0.65 | 0.91 | 0.94 | 0.87 | 1.18 |
|  | Gammaproteobacteria | 0.38 | 0.5 | 0.39 | 0.42 | 0.49 | 0.46 | 0.5 | 0.5 | 0.51 |
|  | Betaproteobacteria | 0.29 | 0.55 | 0.37 | 0.38 | 0.43 | 0.52 | 0.52 | 0.49 | 0.56 |
|  | Bacteroidetes | 0.34 | 0.5 | 0.37 | 0.38 | 0.4 | 0.41 | 0.48 | 0.42 | 0.5 |
|  | Deltaproteobacteria | 0.23 | 0.46 | 0.24 | 0.24 | 0.32 | 0.37 | 0.39 | 0.47 | 0.46 |
|  | Planctomycetes | 0.25 | 0.4 | 0.31 | 0.32 | 0.31 | 0.28 | 0.37 | 0.4 | 0.27 |
|  | Unassigned | 0.14 | 0.28 | 0.16 | 0.17 | 0.2 | 0.21 | 0.26 | 0.24 | 0.25 |
|  | Others | 0.45 | 0.83 | 0.57 | 0.5 | 0.6 | 0.69 | 0.73 | 0.79 | 0.82 |
|  | **Genus (%)** |  |  |  |  |  |  |  |  |  |
| **SPECIALIST** | *Sphingomonas* | 1.96 | 3.94 | 2.72 | 2.24 | 2.68 | 4.39 | 3.67 | 2.96 | 5.63 |
|  | Gp6 | 4.51 | 3.46 | 3.35 | 1.97 | 2.21 | 1.7 | 3.26 | 3.02 | 2 |
|  | *Pseudoxanthomonas* | 0.3 | 0.25 | 0.38 | 6.42 | 1.31 | 1.74 | 1.87 | 1.51 | 1.7 |
|  | *Bacillus* | 0.9 | 0.4 | 0.95 | 2.21 | 2.32 | 2.51 | 0.69 | 1.75 | 1.31 |
|  | *Gemmata* | 1.9 | 1.06 | 1.77 | 0.92 | 1.02 | 0.67 | 1.06 | 1.15 | 0.35 |
|  | *Lysobacter* | 0.67 | 0.92 | 0.49 | 0.74 | 0.91 | 1.19 | 1.26 | 1 | 2.17 |
|  | *Pirellula* | 1.6 | 0.92 | 1.42 | 0.98 | 0.96 | 0.5 | 0.94 | 1.11 | 0.4 |
|  | *Rhizobium* | 0.4 | 0.54 | 0.39 | 1.39 | 1.26 | 1.68 | 1.44 | 0.67 | 1.01 |
|  | *Pseudomonas* | 0.78 | 0.51 | 0.52 | 1.31 | 0.98 | 1.15 | 1.09 | 0.77 | 0.98 |
|  | *Ohtaekwangia* | 1.06 | 1.06 | 1.27 | 0.55 | 1.36 | 0.4 | 0.49 | 0.85 | 0.93 |
|  | *Bradyrhizobium* | 1.07 | 0.89 | 0.96 | 1.4 | 0.69 | 0.45 | 0.59 | 0.81 | 0.52 |
|  | *Povalibacter* | 1.66 | 1 | 1.67 | 0.48 | 0.88 | 0.22 | 0.44 | 0.55 | 0.34 |
|  | *Aridibacter* | 0.37 | 0.76 | 0.63 | 0.49 | 0.5 | 0.48 | 0.81 | 0.69 | 0.7 |
|  | *Devosia* | 0.2 | 0.5 | 0.41 | 0.71 | 0.77 | 0.94 | 0.42 | 0.59 | 0.67 |
|  | *Ensifer* | 0.41 | 0.37 | 0.38 | 0.56 | 0.56 | 0.54 | 0.86 | 0.56 | 0.73 |
|  | Others | 51.65 | 36.26 | 47.84 | 47.11 | 45.77 | 44.78 | 39.5 | 40.37 | 39.76 |
| **OPPORTUNIST** | Gp6 | 3.93 | 5.54 | 3.93 | 3.12 | 3.55 | 2.96 | 5.07 | 4.22 | 3.27 |
|  | *Pirellula* | 0.84 | 0.87 | 0.8 | 0.73 | 0.75 | 0.48 | 0.7 | 0.9 | 0.45 |
|  | *Gemmata* | 0.88 | 0.78 | 0.9 | 0.61 | 0.77 | 0.57 | 0.59 | 0.9 | 0.34 |
|  | *Gemmatimonas* | 0.32 | 0.8 | 0.52 | 0.44 | 0.51 | 0.8 | 0.75 | 0.68 | 0.77 |
|  | Gp4 | 0.32 | 0.72 | 0.52 | 0.42 | 0.52 | 0.57 | 0.86 | 0.66 | 0.63 |
|  | *Neisseria* | 0.56 | 0.57 | 0.52 | 0.53 | 0.54 | 0.6 | 0.59 | 0.57 | 0.57 |
|  | *Zavarzinella* | 0.54 | 0.41 | 0.47 | 0.38 | 0.44 | 0.26 | 0.4 | 0.54 | 0.19 |
|  | *Gaiella* | 0.38 | 0.58 | 0.39 | 0.31 | 0.34 | 0.34 | 0.49 | 0.35 | 0.35 |
|  | *Sphingomonas* | 0.19 | 0.46 | 0.28 | 0.22 | 0.37 | 0.39 | 0.32 | 0.33 | 0.46 |
|  | *Bacillus* | 0.26 | 0.22 | 0.2 | 0.32 | 0.38 | 0.46 | 0.23 | 0.46 | 0.34 |
|  | *Blastopirellula* | 0.41 | 0.45 | 0.37 | 0.34 | 0.32 | 0.15 | 0.29 | 0.34 | 0.14 |
|  | *Mesorhizobium* | 0.23 | 0.35 | 0.31 | 0.26 | 0.29 | 0.38 | 0.31 | 0.27 | 0.35 |
|  | *Ilumatobacter* | 0.3 | 0.44 | 0.27 | 0.22 | 0.25 | 0.24 | 0.27 | 0.25 | 0.25 |
|  | *Steroidobacter* | 0.25 | 0.38 | 0.27 | 0.25 | 0.26 | 0.19 | 0.3 | 0.32 | 0.21 |
|  | Gp3 | 0.18 | 0.35 | 0.21 | 0.21 | 0.26 | 0.27 | 0.23 | 0.31 | 0.34 |
|  | Others | 16.28 | 25.96 | 19.49 | 16.76 | 20.17 | 21.04 | 22.59 | 23.15 | 24.09 |
| **GENERALIST** | Gp6 | 0.24 | 0.48 | 0.31 | 0.28 | 0.31 | 0.35 | 0.48 | 0.42 | 0.38 |
|  | *Veillonella* | 0.18 | 0.2 | 0.21 | 0.17 | 0.2 | 0.16 | 0.19 | 0.2 | 0.18 |
|  | *Haemophilus* | 0.16 | 0.19 | 0.18 | 0.17 | 0.21 | 0.19 | 0.19 | 0.19 | 0.19 |
|  | *Streptococcus* | 0.16 | 0.16 | 0.17 | 0.17 | 0.14 | 0.21 | 0.16 | 0.15 | 0.2 |
|  | *Gaiella* | 0.12 | 0.22 | 0.14 | 0.14 | 0.13 | 0.18 | 0.21 | 0.15 | 0.18 |
|  | *Solirubrobacter* | 0.08 | 0.19 | 0.09 | 0.09 | 0.12 | 0.16 | 0.17 | 0.13 | 0.2 |
|  | *Gemmatimonas* | 0.06 | 0.18 | 0.1 | 0.09 | 0.09 | 0.14 | 0.14 | 0.15 | 0.16 |
|  | *Porphyromonas* | 0.11 | 0.13 | 0.11 | 0.12 | 0.12 | 0.1 | 0.13 | 0.11 | 0.11 |
|  | *Prevotella* | 0.1 | 0.12 | 0.1 | 0.1 | 0.11 | 0.11 | 0.11 | 0.1 | 0.11 |
|  | *Nocardioides* | 0.06 | 0.15 | 0.04 | 0.06 | 0.07 | 0.11 | 0.09 | 0.08 | 0.15 |
|  | *Fusobacterium* | 0.08 | 0.1 | 0.1 | 0.08 | 0.1 | 0.09 | 0.09 | 0.09 | 0.08 |
|  | *Pirellula* | 0.05 | 0.1 | 0.07 | 0.08 | 0.08 | 0.09 | 0.1 | 0.1 | 0.08 |
|  | Gp16 | 0.05 | 0.11 | 0.05 | 0.07 | 0.07 | 0.08 | 0.1 | 0.1 | 0.09 |
|  | *Neisseria* | 0.08 | 0.07 | 0.07 | 0.07 | 0.08 | 0.06 | 0.08 | 0.08 | 0.07 |
|  | *Rothia* | 0.07 | 0.07 | 0.05 | 0.06 | 0.08 | 0.06 | 0.06 | 0.08 | 0.09 |
|  | Others | 3.11 | 5.79 | 3.6 | 3.66 | 4.2 | 4.87 | 5.32 | 5.25 | 5.79 |

**Table S4** Properties of the bacterial cooccurrence networks in different groups

| **Properties of the networks** | CK | L | H |
| --- | --- | --- | --- |
| **Nodes^a^** | 1075 | 655 | 595 |
| **Edges^b^** | 16100 | 1418 | 1229 |
| **Average degree distribution^c^** | 29.953 | 4.33 | 4.131 |
| **Average clustering coefficient^d^** | 0.457 | 0.324 | 0.329 |
| **Average path length^e^** | 3.917 | 6.323 | 6.859 |
| **Modularity^f^** | 0.496 | 0.698 | 0.742 |
| **Modules** | 17 | 82 | 84 |
| **max K-Core** | 35 | 8 | 8 |
| **The proportion of OPPORTUNIST** | 52.84% | 55.11% | 51.26% |
| **The proportion of SPECIALIST** | 30.05% | 26.72% | 31.43% |
| **The proportion of GENERALIST** | 17.11% | 18.17% | 17.31% |

^a^ Number of AVSs with the Spearman correlation |r| > 0.8 and P-value < 0.05. ^b^ Number of significant (P-value < 0.05) correlations between nodes. ^c^ The larger the average distribution, the more complex the network distribution. ^d^ How nodes were embedded in their neighborhood, and the degree to which nodes tend to cluster together. ^e^ The capability of the nodes to form highly connected communities. ^f^ Modularity > 0.4 suggested that the network has a modular structure.

**Table S5** Significant differences in the relative abundance of KEGG function in rhizosphere bacteria at level 2. All values were an average from all replicates ± standard deviations (SD).

| **Level 1** | **Level 2** | **CK** | **L** | **H** | **P value** |
| --- | --- | --- | --- | --- | --- |
| Metabolism | | | | | |
|  | Carbohydrate metabolism | 8.8479±0.1036 a | 8.9958±0.1896 b | 8.9738±0.103 b | 0.0027490 |
|  | Xenobiotics biodegradation and metabolism | 4.0379±0.1886 b | 3.7325±0.3712 a | 3.9047±0.1883 a | 0.0045369 |
|  | Energy metabolism | 3.4194±0.0596 b | 3.3787±0.0654 a | 3.3767±0.0405 a | 0.0347493 |
|  | Biosynthesis of other secondary metabolites | 1.3807±0.0282 a | 1.4195±0.0469 b | 1.411±0.0306 b | 0.0037556 |
| Human Diseases | | | | | |
|  | Infectious diseases: Viral | 0.1756±0.0062 b | 0.1707±0.0183 b | 0.1651±0.0079 a | 0.0008360 |
|  | Endocrine and metabolic diseases | 0.154±0.0046 b | 0.1487±0.0034 a | 0.1479±0.0033 a | 0.0001756 |
|  | Substance dependence | 0.0636±0.0031 b | 0.051±0.0102 a | 0.0538±0.0055 a | 0.0000005 |
|  | Infectious diseases: Parasitic | 0.062±0.0025 a | 0.0657±0.0048 b | 0.0635±0.0022 a | 0.0303639 |
|  | Immune diseases | 0.0174±0.0017 a | 0.0189±0.0032 b | 0.0195±0.003 b | 0.0335686 |
| Organismal Systems | | | | | |
|  | Endocrine system | 0.6527±0.0356 b | 0.6133±0.0655 a | 0.649±0.0342 b | 0.0424334 |
|  | Environmental adaptation | 0.2437±0.0082 b | 0.2387±0.0132 a | 0.2475±0.0086 b | 0.0499886 |
|  | Nervous system | 0.128±0.0023 b | 0.1224±0.0084 a | 0.1229±0.0045 a | 0.0002692 |
|  | Immune system | 0.0893±0.0071 a | 0.0938±0.0095 b | 0.0922±0.0076 b | 0.0450644 |
|  | Development | 0.0004±0.0003 a | 0.0006±0.0002 b | 0.0004±0.0001 a | 0.0024737 |
| Genetic Information Processing | | | | | |
|  | Replication and repair | 1.0863±0.0322 a | 1.1428±0.0909 b | 1.1253±0.036 b | 0.0009583 |
|  | Folding, sorting and degradation | 0.716±0.0285 a | 0.7372±0.046 c | 0.7238±0.0184 b | 0.0354028 |
|  | Transcription | 0.0594±0.0013 a | 0.0618±0.0039 b | 0.0613±0.0022 b | 0.0014558 |

Note: Statistical significance was assessed by one-way ANOVA followed by Tukey HSD test.

**Table S6** Differences of plant growth parameters. All values were an average from all replicates ± standard deviations (SD).

| **Plant growth parameters** | **CK** | **L** | **H** | **P value** |
| --- | --- | --- | --- | --- |
| Plant height (cm) | 10.150 ± 1.287 a | 14.630 ± 1.465 b | 17.820 ± 2.404 c | ** |
| Number of leaves | 7.500 ± 1.581 a | 8.000 ± 1.563 a | 8.600 ± 1.265 a | ns |
| Maximum leaf length (cm) | 9.710 ± 1.308 a | 14.240 ± 1.657 b | 17.490 ± 2.523 c | ** |
| Maximum leaf width (cm) | 4.610 ± 0.874 a | 5.990 ± 0.994 b | 7.350 ± 1.023 c | ** |
| Root length (cm) | 7.810 ± 1.112 a | 9.470 ± 1.993 a | 9.110 ± 1.455 a | ns |
| Dry weight (g) | 0.771 ± 0.238 a | 1.438 ± 0.449 b | 2.126 ± 0.322 c | ** |

Note: Statistical significance was assessed by one-way ANOVA followed by Tukey HSD test.

**Table S7** Soil physicochemical properties in the study.

| **Sample** | **Days** | **SOM**  **(g/kg)** | **TN**  **(g/kg)** | **AN**  **(mg/kg)** | **TK**  **(g/kg)** | **AK**  **(mg/kg)** | **SK**  **(mg/kg)** | **TP**  **(g/kg)** | **AP**  **(mg/kg)** | **Cu**  **(mg/kg)** | **Zn**  **(mg/kg)** | **Fe**  **(g/kg)** | **Mn**  **(g/kg)** | **EC**  **(μs/cm)** | **pH** |
| --- | --- | --- | --- | --- | --- | --- | --- | --- | --- | --- | --- | --- | --- | --- | --- |
| CK1_1 | 2 | 26.75 | 1.32 | 77.80 | 18.92 | 365 | 1242 | 0.80 | 29.61 | 20.46 | 71.51 | 26.99 | 0.52 | 212.5 | 8.2 |
| CK1_2 | 2 | 27.41 | 1.35 | 78.98 | 17.78 | 355 | 1202 | 0.91 | 32.78 | 25.39 | 60.91 | 30.21 | 0.54 | 226.8 | 8.1 |
| CK1_3 | 2 | 25.03 | 1.21 | 85.51 | 20.31 | 375 | 1185 | 0.87 | 32.59 | 20.78 | 73.91 | 27.37 | 0.55 | 213.4 | 8.1 |
| CK1_4 | 2 | 24.25 | 1.22 | 76.48 | 17.54 | 360 | 1264 | 0.84 | 32.15 | 19.34 | 70.74 | 27.64 | 0.49 | 209.2 | 8.2 |
| CK1_5 | 2 | 25.58 | 1.27 | 76.51 | 18.57 | 370 | 1223 | 0.76 | 31.85 | 19.54 | 69.29 | 26.81 | 0.53 | 215.0 | 8.3 |
| CK1_6 | 2 | 26.50 | 1.26 | 81.44 | 19.69 | 339 | 1243 | 0.95 | 34.49 | 19.85 | 65.74 | 24.31 | 0.53 | 210.0 | 8.0 |
| CK2_1 | 16 | 28.04 | 1.41 | 66.53 | 18.86 | 394 | 1242 | 0.81 | 31.66 | 20.46 | 74.76 | 28.30 | 0.52 | 214.2 | 8.6 |
| CK2_2 | 16 | 33.53 | 1.39 | 66.95 | 18.01 | 396 | 1293 | 0.80 | 34.07 | 18.64 | 68.83 | 31.99 | 0.54 | 216.7 | 8.4 |
| CK2_3 | 16 | 31.55 | 1.29 | 64.52 | 20.30 | 370 | 1282 | 0.94 | 32.25 | 20.55 | 79.40 | 32.27 | 0.52 | 215.9 | 8.5 |
| CK2_4 | 16 | 29.04 | 1.30 | 68.94 | 17.89 | 416 | 1228 | 0.81 | 33.15 | 21.11 | 71.29 | 40.89 | 0.51 | 220.9 | 8.6 |
| CK2_5 | 16 | 29.26 | 1.22 | 63.85 | 20.39 | 394 | 1257 | 0.92 | 31.17 | 19.50 | 76.19 | 32.00 | 0.52 | 212.5 | 8.6 |
| CK2_6 | 16 | 29.59 | 1.28 | 70.36 | 19.80 | 389 | 1222 | 0.91 | 31.75 | 21.32 | 80.18 | 33.61 | 0.53 | 211.7 | 8.5 |
| CK3_1 | 32 | 34.76 | 1.32 | 69.22 | 18.56 | 384 | 1312 | 1.02 | 31.97 | 19.90 | 74.29 | 28.02 | 0.52 | 154.6 | 8.7 |
| CK3_2 | 32 | 31.34 | 1.53 | 69.04 | 19.40 | 410 | 1357 | 1.16 | 31.66 | 22.47 | 76.95 | 27.28 | 0.54 | 157.1 | 8.6 |
| CK3_3 | 32 | 29.78 | 1.47 | 69.54 | 17.08 | 398 | 1317 | 0.84 | 33.94 | 19.61 | 71.56 | 22.29 | 0.51 | 142.8 | 8.7 |
| CK3_4 | 32 | 28.51 | 1.42 | 73.81 | 18.38 | 379 | 1345 | 1.09 | 36.19 | 22.06 | 79.69 | 26.20 | 0.54 | 164.6 | 8.6 |
| CK3_5 | 32 | 30.06 | 1.48 | 67.58 | 19.72 | 435 | 1335 | 0.94 | 33.32 | 19.60 | 70.42 | 28.30 | 0.52 | 158.8 | 8.7 |
| CK3_6 | 32 | 34.21 | 1.41 | 71.85 | 19.03 | 405 | 1346 | 1.10 | 34.73 | 19.59 | 72.71 | 25.29 | 0.52 | 158.8 | 8.7 |
| L1_1 | 2 | 28.16 | 1.32 | 94.08 | 19.92 | 485 | 1524 | 1.02 | 48.50 | 21.49 | 75.33 | 29.82 | 0.53 | 239.2 | 8.4 |
| L1_2 | 2 | 27.49 | 1.46 | 88.59 | 19.74 | 480 | 1539 | 1.11 | 52.59 | 24.78 | 79.24 | 28.76 | 0.52 | 240.1 | 8.4 |
| L1_3 | 2 | 26.09 | 1.39 | 88.40 | 20.58 | 477 | 1550 | 0.91 | 44.40 | 27.27 | 82.14 | 30.60 | 0.56 | 232.8 | 8.2 |
| L1_4 | 2 | 29.89 | 1.34 | 97.94 | 20.37 | 453 | 1536 | 1.09 | 48.65 | 23.17 | 73.23 | 33.29 | 0.57 | 230.0 | 8.4 |
| L1_5 | 2 | 29.4 | 1.37 | 89.27 | 19.47 | 445 | 1615 | 1.02 | 53.94 | 25.84 | 83.31 | 21.83 | 0.51 | 255.2 | 8.3 |
| L1_6 | 2 | 26.34 | 1.27 | 95.82 | 20.25 | 469 | 1513 | 0.97 | 46.78 | 21.93 | 74.99 | 32.52 | 0.55 | 230.7 | 8.2 |
| L2_1 | 16 | 33.58 | 1.57 | 110.99 | 20.19 | 520 | 1500 | 1.06 | 52.75 | 22.68 | 72.71 | 29.74 | 0.55 | 274.5 | 8.6 |
| L2_2 | 16 | 31.31 | 1.46 | 105.51 | 19.74 | 502 | 1541 | 0.99 | 58.84 | 25.75 | 78.09 | 32.22 | 0.55 | 282.5 | 8.5 |
| L2_3 | 16 | 33.92 | 1.52 | 103.16 | 21.02 | 483 | 1512 | 1.21 | 53.82 | 20.49 | 81.95 | 30.51 | 0.53 | 276.6 | 8.6 |
| L2_4 | 16 | 36.59 | 1.61 | 112.83 | 19.39 | 532 | 1457 | 0.85 | 51.69 | 20.23 | 70.45 | 34.84 | 0.54 | 265.4 | 8.5 |
| L2_5 | 16 | 34.81 | 1.64 | 109.76 | 20.77 | 547 | 1575 | 1.33 | 57.27 | 21.99 | 74.53 | 31.16 | 0.49 | 266.0 | 8.6 |
| L2_6 | 16 | 33.69 | 1.54 | 112.19 | 18.91 | 536 | 1505 | 1.27 | 52.07 | 21.90 | 72.96 | 34.78 | 0.55 | 282.5 | 8.6 |
| L3_1 | 32 | 33.86 | 1.46 | 100.70 | 20.47 | 470 | 1516 | 1.06 | 52.50 | 22.41 | 97.18 | 30.55 | 0.60 | 204.9 | 8.7 |
| L3_2 | 32 | 33.56 | 1.52 | 100.57 | 20.43 | 504 | 1523 | 1.17 | 51.78 | 23.22 | 93.01 | 38.56 | 0.56 | 223.4 | 8.7 |
| L3_3 | 32 | 35.86 | 1.44 | 106.32 | 19.50 | 480 | 1566 | 1.16 | 50.89 | 22.50 | 96.5 | 28.88 | 0.57 | 196.4 | 8.7 |
| L3_4 | 32 | 33.15 | 1.45 | 99.09 | 20.39 | 467 | 1543 | 1.30 | 52.49 | 25.15 | 102.66 | 34.36 | 0.59 | 226.1 | 8.7 |
| L3_5 | 32 | 34.35 | 1.42 | 95.21 | 19.18 | 473 | 1526 | 1.03 | 52.58 | 23.04 | 89.19 | 36.22 | 0.61 | 219.5 | 8.8 |
| L3_6 | 32 | 37.61 | 1.41 | 109.74 | 21.24 | 455 | 1546 | 1.34 | 49.37 | 24.85 | 94.15 | 21.68 | 0.59 | 194.0 | 8.7 |
| H1_1 | 2 | 29.57 | 1.26 | 92.49 | 21.37 | 644 | 1541 | 1.04 | 43.56 | 22.54 | 76.28 | 33.97 | 0.58 | 212.1 | 8.5 |
| H1_2 | 2 | 29.08 | 1.25 | 78.66 | 20.50 | 647 | 1561 | 0.91 | 45.43 | 20.53 | 79.46 | 32.92 | 0.59 | 212.1 | 8.6 |
| H1_3 | 2 | 29.78 | 1.37 | 90.23 | 20.66 | 632 | 1458 | 1.14 | 42.85 | 22.66 | 75.19 | 37.42 | 0.65 | 213.2 | 8.6 |
| H1_4 | 2 | 31.33 | 1.70 | 80.77 | 19.82 | 660 | 1503 | 1.00 | 47.61 | 22.16 | 77.03 | 32.27 | 0.62 | 213.2 | 8.6 |
| H1_5 | 2 | 30.91 | 1.59 | 95.77 | 22.43 | 613 | 1584 | 1.21 | 41.77 | 25.43 | 75.87 | 31.66 | 0.59 | 207.9 | 8.6 |
| H1_6 | 2 | 33.06 | 1.31 | 88.25 | 19.44 | 638 | 1478 | 1.19 | 44.48 | 24.97 | 75.94 | 36.51 | 0.57 | 214.2 | 8.6 |
| H2_1 | 16 | 38.94 | 1.56 | 108.50 | 21.16 | 540 | 1467 | 1.18 | 49.32 | 23.08 | 74.43 | 33.00 | 0.57 | 228.9 | 8.5 |
| H2_2 | 16 | 40.45 | 1.50 | 109.32 | 19.4 | 551 | 1443 | 1.19 | 50.02 | 22.00 | 77.28 | 36.74 | 0.57 | 223.7 | 8.5 |
| H2_3 | 16 | 38.78 | 2.04 | 105.43 | 21.65 | 534 | 1461 | 1.53 | 48.11 | 20.68 | 77.68 | 32.35 | 0.62 | 232.1 | 8.6 |
| H2_4 | 16 | 39.81 | 1.72 | 103.81 | 20.50 | 545 | 1429 | 1.36 | 47.50 | 21.62 | 71.51 | 30.55 | 0.63 | 241.5 | 8.4 |
| H2_5 | 16 | 37.24 | 1.40 | 113.06 | 22.05 | 521 | 1447 | 1.45 | 54.02 | 28.76 | 74.47 | 30.95 | 0.53 | 238.4 | 8.5 |
| H2_6 | 16 | 37.92 | 1.79 | 105.93 | 20.50 | 531 | 1490 | 1.08 | 45.34 | 23.69 | 80.22 | 37.87 | 0.68 | 232.1 | 8.4 |
| H3_1 | 32 | 35.05 | 1.55 | 105.83 | 21.20 | 511 | 1495 | 1.16 | 49.68 | 22.55 | 78.16 | 32.21 | 0.57 | 195.3 | 8.6 |
| H3_2 | 32 | 35.84 | 1.43 | 107.40 | 20.56 | 536 | 1493 | 1.17 | 53.17 | 23.69 | 74.15 | 32.22 | 0.58 | 194.3 | 8.8 |
| H3_3 | 32 | 40.27 | 1.51 | 113.12 | 20.10 | 499 | 1485 | 1.17 | 50.86 | 25.19 | 86.3 | 36.13 | 0.55 | 193.2 | 8.7 |
| H3_4 | 32 | 36.04 | 1.49 | 104.82 | 23.05 | 466 | 1497 | 1.18 | 50.07 | 28.57 | 71.38 | 34.35 | 0.56 | 193.2 | 8.7 |
| H3_5 | 32 | 33.48 | 1.76 | 115.95 | 21.44 | 482 | 1501 | 1.3 | 51.80 | 25.25 | 86.38 | 30.29 | 0.55 | 201.6 | 8.6 |
| H3_6 | 32 | 36.16 | 1.66 | 114.23 | 22.01 | 559 | 1439 | 1.22 | 49.37 | 23.86 | 79.81 | 31.8 | 0.54 | 207.9 | 8.6 |
